# ﻿Comparative mitogenomic analysis of two earwigs (Insecta, Dermaptera) and the preliminary phylogenetic implications

**DOI:** 10.3897/zookeys.1087.78998

**Published:** 2022-02-23

**Authors:** Zhi-Teng Chen

**Affiliations:** 1 School of Grain Science and Technology, Jiangsu University of Science and Technology, Zhenjiang 212004, China Jiangsu University of Science and Technology Zhenjiang China

**Keywords:** Apachyidae, Dermaptera, Diplatyidae, mitochondrial genome, phylogeny

## Abstract

The phylogenetic position and inner relationships of Dermaptera remain unresolved despite the numerous efforts using morphological and molecular data. To facilitate the resolution of problems, this study sequenced the complete mitogenome of *Apachyusfeae* de Bormans, 1894 (Apachyidae) and the nearly complete mitogenome of *Diplatysflavicollis* Shiraki, 1907 (Diplatyidae). The 19,029-bp long mitogenome of *A.feae* exhibited an extra *trnV* gene and two control regions in addition to the typical set of 37 genes including 13 protein-coding genes (PCGs), 22 transfer RNA (tRNA) genes, and two ribosomal RNA (rRNA) genes. The 12,950-bp long partially sequenced mitogenome of *D.flavicollis* was composed of 10 and a partial fragment of PCGs, 18 tRNA genes, two rRNA genes, and a control region. Comparative analysis of available earwig mitogenomes revealed variable mitogenomic structure and extensive gene rearrangements in Dermaptera. The preliminary phylogenetic analyses using Bayesian inference and maximum likelihood methods showed identical results, but the limited sampling and different types of molecular data lead to an apparent incongruence with previous phylogenetic studies.

## ﻿Introduction

Dermaptera (earwigs) are a small group of ancient insects in Polyneoptera, with more than 1900 extant species within 11 families known worldwide (Haas 2018). The characteristics such as forceps‐like, unsegmented cerci in the adults of this group are functional in predation, defense, wingfolding and mating (Haas et al. 2000). Most earwigs are free-living and commonly found in damp areas feeding on plant materials, spores, fungi, or insects (Haas 2018). With the exception of Arixeniidae and Hemimeridae, these two families are distinctly epizoic and live non‐parasitically on cavernicolous bats and hamster rats, respectively (Nakata and Maa 1974; Haas and Gorb 2004). The majority of earwigs are oviparous, whereas the epizoic groups are viviparous, i.e., directly giving birth to nymphs. Besides, unusual maternal care behavior is found in all studied earwig species, with the female protecting eggs and first‐instar nymphs (Suzuki et al. 2005; Staerkle and Koelliker 2008).

The extant Dermaptera is traditionally divided into three suborders, i.e., Arixeniina, Hemimerina, and Forficulina (Gullan and Cranston 2010). Arixeniidae and Hemimeridae are sometimes considered to be derived members of Forficulina (nonparasitic Dermaptera) in several studies (Popham 1985; Klass 2001; Engel and Haas 2007). The most recent reclassification of Dermaptera was established by Engel and Haas (2007), which included all extant earwigs in the suborder Neodermaptera. Protodermaptera and Epidermaptera are recognized as two infraorders in Neodermaptera, and Epidermaptera comprises the two epizoic families.

The phylogenetic position of Dermaptera in Insecta and the inner relationship within Dermaptera remain controversial (Beutel et al. 2013). Different research using morphological characteristics or molecular data from nuclear and mitochondrial genes generated different phylogenies of Dermaptera (Wan et al. 2012; Naegle et al. 2016). Wan et al. (2012) sequenced and analyzed the first earwig mitochondrial genome (mitogenome) and investigated the phylogeny of Polyneoptera. To date, *Challiafletcheri* Burr, 1904 and *Euborelliaarcanum* Matzke & Kocarek, 2015 are the only two complete earwig mitogenomes available in GenBank, and only the mitogenomic structure of *C.fletcheri* has been analyzed (Wan et al. 2012). To better resolve the phylogeny of earwigs using mitogenomic data, this study sequenced and analyzed two new mitogenomes for Dermaptera. A preliminary phylogenetic tree of Dermaptera is constructed based on the newly sequenced and the known mitogenomic data to provide a basic topology for the relationships among families.

## ﻿Materials and methods

### ﻿Sample preparation and DNA extraction

The specimen of *Apachyusfeae* de Bormans, 1894 was collected from Laibin, Guangxi Province of China (24.1402°N, 110.1844°E) in October of 2019; the specimen of *Diplatysflavicollis* Shiraki, 1907 was collected from Jurong, Jiangsu Province of China (32.1325°N, 119.0743°E) in February of 2020. The specimens were identified by the author, preserved in 100% ethanol, and stored in the Insect Collection of Jiangsu University of Science and Technology (**ICJUST**). The total genomic DNA of the two earwigs was isolated using the E.Z.N.A. Tissue DNA Kit (Omega Bio-Tek, Inc.) and preserved at −20 °C before the sequencing process.

### ﻿Sequencing, assembly, annotation, and analysis

The Illumina TruSeq short-insert libraries (size = 450 bp) were constructed using 1 μg of purified DNA fragments and were sequenced by Illumina Hiseq 4000 (Shanghai Biozeron Biotechnology Co., Ltd). Raw reads were filtered prior to assembly; high-quality reads were retained and assembled into contigs by SOAPdenovo2.04 (Luo et al. 2012). The assembled contigs were then aligned to the reference mitogenomes of Dermaptera using BLAST. Subsequently, the aligned contigs (≥80% similarity and query coverage) were arranged according to the reference mitogenomes. Finally, the clean reads were mapped to the assembled draft mitogenomes to fix the wrong bases; gaps were filled using GapFiller v. 2.1.1 (https://sourceforge.net/projects/gapfiller/). The mitogenome sequences of *A.feae* and *D.flavicollis* were deposited in GenBank under the accession numbers MW291948 and MW291949, respectively. Mitochondrial gene analyses of *A.feae* and *D.flavicollis* were compared to four additional species of Dermaptera with available mitogenomes (Table 1). The gene order was compared with *Drosophilayakuba* Burla, 1954, which was considered to possess the ancestral arthropod mitochondrial gene arrangement (Clary and Wolstenholme 1985).

**Table 1.** List of species used in this study.

Infraorder	Parvorder	Family	Species	Length (bp)	A+T%	Accession number
Protodermaptera		Diplatyidae	*Diplatysflavicollis	12,950	73.5	MW291949
		Pygidicranidae	*Challiafletcheri*	20,456	72.6	NC_018538
Epidermaptera	Paradermaptera	Apachyidae	*Apachyusfeae*	19,029	61.2	MW291948
	Metadermaptera	Anisolabididae	*Euborelliaarcanum*	16,087	68.3	KX673196
	Eteodermaptera	Forficulidae	*Eudohrniametallica	16,324	58.7	KX091853
			*Paratimomenusflavocapitatus	15,677	67.4	KX091861
Outgroup	Outgroup	Outgroup	*Kamimuriachungnanshana*	—	—	NC_028076

* Incomplete mitogenomes.

All protein-coding genes (**PCGs**) and ribosomal RNA (**rRNA**) genes were identified by homology alignments. Gene boundaries of each PCG were further confirmed by ORF finder (https://www.ncbi.nlm.nih.gov/orffinder/). All transfer RNA (**tRNA**) genes were predicted and illustrated by the MITOS online server (Bernt et al. 2013). The visual structure of the mitogenomes were depicted using CGView Server (http://stothard.afns.ualberta.ca/cgview_server/) (Grant and Stothard 2008). Nucleotide composition of each gene and codon usage of PCGs were calculated using MEGA v. 6.0 (Tamura et al. 2013). The composition skew values were calculated by AT-skew = [A – T] / [A + T] and GC-skew = [G – C] / [G + C] formulas (Perna and Kocher 1995). The synonymous substitution rate (Ks) and nonsynonymous substitution rate (Ka) were computed by DnaSP v. 5.10 (Librado and Rozas 2009). Presumed secondary structures in the control regions were predicted by the online tool Tandem Repeats Finder (http://tandem.bu.edu/trf/trf.advanced.submit.html), DNAMAN v. 6.0.3 and ARWEN (http://mbio-serv2.mbioekol.lu.se/ARWEN/) (Laslett and Canbäck 2008).

### ﻿Phylogenetic analysis

Nucleotide sequences of PCGs derived from six species of Dermaptera, including *A.feae* and *D.flavicollis* sequenced in this study, were used in the phylogenetic analysis (Table 1). The stonefly *Kamimuriachungnanshana* Wu, 1938 (Plecoptera, Perlidae; GenBank accession no. NC_028076) was used as the outgroup. The 13 PCGs were respectively aligned by MAFFT and concatenated as a combined dataset using SequenceMatrix v. 1.7.8 (Katoh and Standley 2013). The optimal nucleotide substitution models and partitioning schemes for the dataset was determined by PartitionFinder v. 2.1.1 using the Bayesian Information Criterion (BIC) and a greedy search algorithm (Lanfear et al. 2016). Bayesian inferences (BI) and Maximum likelihood (ML) analyses were conducted with the optimal partition schemes. The BI analysis was conducted by MrBayes v. 3.2.7, with 20 million generations sampling every 1000 generations, running one cold chain and three hot chains with a burn-in of 25% trees (Ronquist and Huelsenbeck 2003). TRACER v. 1.5 was used to examine the stability of the results of the BI analysis. The ML analysis was performed by RAxML v. 8.2.12 with 1000 bootstrap replicates (Stamatakis 2014). FigTree v. 1.4.2 was used to adjust and visualize the tree files generated by both BI and ML inferences.

## ﻿Results

### ﻿Mitogenome annotation and nucleotide composition

**Figure 1.**
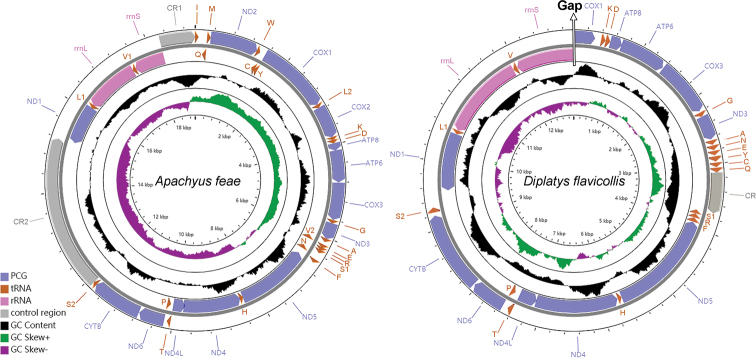
Mitochondrial maps of *Apachyusfeae* and *Diplatysflavicollis*. Genes outside the map are transcribed clockwise, whereas those inside the map are transcribed counterclockwise. Names and other details of the genes are listed in Tables 2 and 3. The inside circles show the GC content and the GC skew. GC content and GC skew are plotted as the deviation from the average value of the entire sequence.

The complete mitogenome of *A.feae* is a typical double-strand circular molecule with a length of 19,029 bp (Fig. 1). The obtained partial mitogenome of *D.flavicollis* is 12,950 bp in length (Fig. 1). The completely sequenced three mitogenomes of Dermaptera range in size from 16,087 bp in *E.arcanum* to 20,456 bp in *C.fletcheri*. In the mitogenome of *A.feae*, an extra *trnV* gene and two control regions are found in addition to the standard set of 37 genes (13 PCGs, 22 tRNA genes and two rRNA genes) (Table 2). In the partial mitogenome of *D.flavicollis*, 10 and a partial fragment of PCGs, 18 tRNA genes, two rRNA genes, and a control region are annotated (Table 3). In *A.feae*, there are 56 overlapping nucleotides located in three pairs of neighboring genes, and the longest overlap is 41-bp long and located between *trnT* and *ND4L* (Table 2). A total of 296 intergenic nucleotides (IGNs) are dispersed in 19 locations for *A.feae*. In *D.flavicollis*, 17 overlapping nucleotides and 504 IGNs are found, including a 227-bp long IGN between *trnS2* (*UCN*) and *ND1* (Table 3).

**Table 2.** Mitochondrial genome structure of *Apachyusfeae*.

Gene	Position (bp)	Size (bp)	Direction	Intergenic nucleotides	Anti- or start/stop codons	A+T%
*trnIle* (*I*)	1–62	62	Forward	0	GAT	64.5
*trnGln* (*Q*)	171–240	70	Reverse	108	TTG	65.7
*trnMet* (*M*)	257–326	70	Forward	16	CAT	61.4
*ND2*	328–1347	1020	Forward	1	ATT/TAA	62.3
*trnTrp* (W)	1350–1415	66	Forward	2	TCA	63.6
*trnCys* (*C*)	1408–1474	67	Reverse	−8	GCA	62.7
*trnTyr* (*Y*)	1476–1539	64	Reverse	1	GTA	67.2
*COX1*	1540–3075	1536	Forward	0	ATG/TAG	58.1
*trnL2* (*UUR*)	3081–3147	67	Forward	5	TAA	62.7
*COX2*	3148–3831	684	Forward	0	ATG/TAG	58.0
*trnLys* (*K*)	3832–3901	70	Forward	0	CTT	61.4
*trnAsp* (*D*)	3903–3971	69	Forward	1	GTC	79.7
*ATP8*	3972–4133	162	Forward	0	GTG/TAG	57.4
*ATP6*	4127–4807	681	Forward	−7	ATG/TAG	58.1
*COX3*	4813–5607	795	Forward	5	TTG/TAA	56.7
*trnGly* (*G*)	5620–5680	61	Forward	12	TCC	78.7
*ND3*	5681–6034	354	Forward	0	ATG/TAG	56.8
*trnAla* (*A*)	6036–6099	64	Forward	1	TGC	45.3
*trnVal2* (*GUU*)	6109–6168	60	Reverse	9	AAC	60.0
*trnGlu* (*E*)	6177–6238	62	Forward	8	TTC	74.2
*trnArg* (*R*)	6241–6301	61	Forward	2	TCG	68.9
*trnSer1* (*AGN*)	6303–6363	61	Forward	1	GCT	70.5
*trnAsn* (*N*)	6385–6448	64	Reverse	21	GTT	58.5
*trnPhe* (*F*)	6533–6598	66	Forward	84	GAA	77.3
*ND5*	6599–8347	1749	Reverse	0	ATG/TAA	57.5
*trnHis* (*H*)	8348–8414	67	Reverse	0	GTG	61.2
*ND4*	8415–9795	1381	Reverse	0	ATG/T−	59.9
*ND4L*	9755–10045	291	Reverse	−41	ATG/TAA	60.8
*trnThr* (*T*)	10,053–10,115	63	Forward	7	TGT	73.0
*trnPro* (*P*)	10,116–10,179	64	Reverse	0	TGG	64.1
*ND6*	10,182–10,730	549	Forward	2	ATT/TAA	62.8
*CYTB*	10,741–11,818	1078	Forward	10	ATT/T–	58.2
*trnSer2* (*UCN*)	11,819–11,887	69	Forward	0	TGA	73.9
*CR2*	11,888–15,172	3285	Forward	0	—	59.5
*ND1*	15,173–16,120	948	Reverse	0	ATG/TAG	62.3
*trnLeu1* (*CUN*)	16,121–16,187	67	Reverse	0	TAG	70.1
*rrnL*	16,188–17,467	1280	Reverse	0	—	67.9
*trnV1* (*GUA*)	17,468–17,534	67	Reverse	0	TAC	67.2
*rrnS*	17,535–18,273	739	Reverse	0	—	66.0
*CR1*	18,274–19,029	756	Forward	0	—	74.2

**Table 3.** Mitochondrial genome structure of *Diplatysflavicollis*.

Gene	Position (bp)	Size (bp)	Direction	Intergenic nucleotides	Anti- or start/stop codons	A+T%
*COX1* (*partial*)	1–310	310	Forward	0	?/TAA	64.5
*trnLys* (*K*)	398–463	66	Forward	87	CTT	68.2
*trnAsp* (*D*)	464–532	69	Forward	0	GTC	87.0
*ATP8*	533–706	174	Forward	0	ATT/TAG	75.9
*ATP6*	700–1377	678	Forward	−7	ATG/TAA	72.5
*COX3*	1388–2200	813	Forward	10	ATT/TAA	68.5
*trnGly* (*G*)	2222–2286	65	Forward	21	TCC	75.4
*ND3*	2287–2637	351	Forward	0	ATT/TAA	74.3
*trnAla* (*A*)	2660–2724	65	Forward	22	TGC	77.6
*trnAsn* (*N*)	2736–2803	68	Forward	11	GTT	78.5
*trnGlu* (*E*)	2815–2879	65	Forward	11	TTC	77.5
*trnTyr* (*Y*)	2895–2969	75	Forward	15	GTA	80.0
*trnCys* (*C*)	2985–3051	67	Forward	15	GCA	79.7
*trnGln* (*Q*)	3059–3127	69	Forward	7	TTG	76.8
CR	3128–3719	592	Forward	0	—	82.6
*trnSer1* (*AGN*)	3720–3784	65	Reverse	0	GCT	69.2
*trnArg* (*R*)	3785–3852	68	Reverse	0	TCG	78.3
*trnPhe* (*F*)	3854–3925	72	Reverse	1	GAA	90.5
*ND5*	3928–5673	1746	Reverse	2	ATC/TAA	71.5
*trnHis* (*H*)	5674–5739	66	Reverse	0	GTG	83.6
*ND4*	5747–7099	1353	Reverse	7	ATC/TAA	72.0
*ND4L*	7090–7386	297	Reverse	−10	ATT/TAA	74.3
*trnThr* (*T*)	7394–7464	71	Forward	7	TGT	74.6
*trnPro* (*P*)	7465–7537	73	Reverse	0	TGG	80.0
*ND6*	7540–8043	504	Forward	2	ATT/TAG	76.4
*CYTB*	8056–9198	1143	Forward	12	ATG/TAG	69.8
*trnSer2* (*UCN*)	9246–9318	73	Forward	47	TGA	77.0
*ND1*	9546–10487	942	Reverse	227	ATT/TAA	69.9
*trnLeu1* (*CUN*)	10,488–10,554	67	Reverse	0	TAG	79.1
*rrnL*	10,555–11,918	1364	Reverse	0	—	76.1
*trnVal* (*V*)	11,919–11,990	72	Reverse	0	TAC	72.2
*rrnS*	11,991–12,950	960	Reverse	0	—	76.9

The mitogenomes of *A.feae* and *D.flavicollis* are biased toward A and T nucleotides (61.2% and 73.5%, respectively), which is consistent with other earwigs (Table 1). The A+T contents were also rich in each mitochondrial gene, showing the highest in *trnD* of *A.feae* and *trnF* of *D.flavicollis*.

### ﻿Gene rearrangement

**Figure 2.**
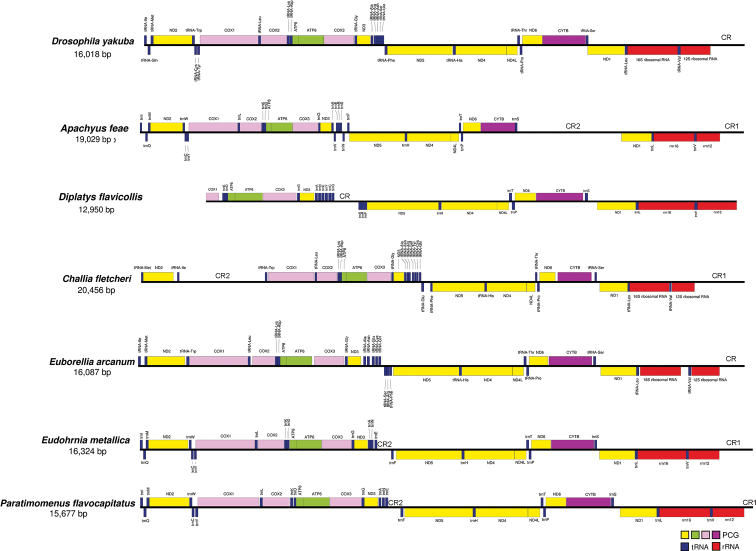
Mitochondrial gene arrangement of six earwigs in comparison with *Drosophilayakuba*.

In the sequenced earwigs, no PCG rearrangement are found (Fig. 2). In *A.feae*, most tRNA genes in the gene cluster *trnA*-*R*-*N*-*S1*-*E*-*F* are rearranged, and an extra *trnV* is present in the gene cluster. In *D.flavicollis*, the gene cluster *trnA*-*R*-*N*-*S1*-*E*-*F* is also rearranged and incorporates *trnY*, *trnC* and *trnQ* from other locations. In *C.fletcheri*, *trnI*, *trnC*, *trnY*, *trnQ*, and *trnE* are rearranged (Wan et al. 2012). In *E.arcanum*, *trnQ*, *trnC*, *trnY*, *trnR*, and *trnS1* are rearranged, and *trnY* is lost. In *E.metallica* and *P.flavocapitatus*, both *trnR* and *trnS1* are absent. These tRNA rearrangements mainly occur in the *trnA*-*R*-*N*-*S1*-*E*-*F* gene cluster. The two rRNA genes are located in the same location for all sequenced earwigs; however, they are variable in size interspecifically. In addition to the tRNA rearrangements, the control region of *D.flavicollis* transfers to the new location between *ND3* and *ND5*; an extra control region is also found in *A.feae* and *C.fletcheri* (Wan et al. 2012).

### ﻿Protein-coding genes (PCGs)

**Figure 3.**
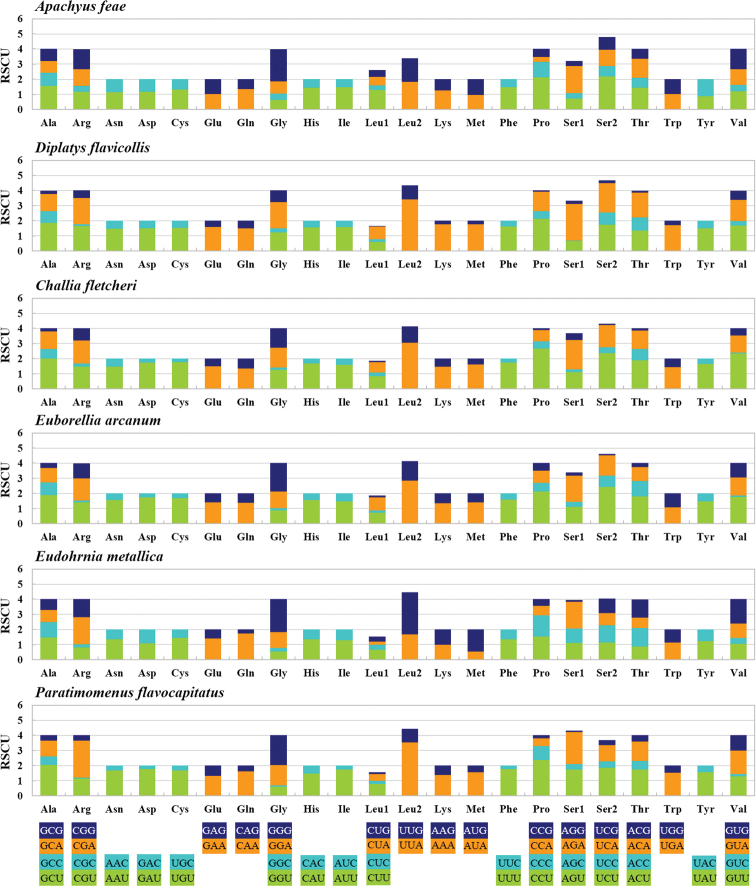
Relative synonymous codon usage (RSCU) of PCGs in six species of earwigs.

All PCGs of *A.feae* are annotated, whereas *ND2*, *COX2*, and partial *COX1* of *D.flavicollis* are not sequenced. The PCGs of *A.feae* are similar in size to those of *D.flavicollis* and other earwigs. Most PCGs of *A.feae* and all PCGs of *D.flavicollis* utilize the standard ATN start codon (ATT, ATC, and ATG), whereas *ATP8* and *COX3* of *A.feae* start with special start codons (GTG and TTG, respectively) (Tables 2, 3). Most PCGs of *A.feae* and all PCGs of *D.flavicollis* have the complete termination codon TAN (TAA or TAG), whereas *ND4* and *CYTB* of *A.feae* end with an incomplete stop codon T (Tables 2, 3). The relative synonymous codon usage (RSCU) values were calculated for the six earwig mitogenomes (Fig. 3). The most frequently used codon is TCT (Ser) for *A.feae*, TTG (Leu) for *E.metallica*, TTA (Leu) for *D.flavicollis*, *C.fletcheri*, *E.arcanum*, and *P.flavocapitatus*.

**Figure 4.**
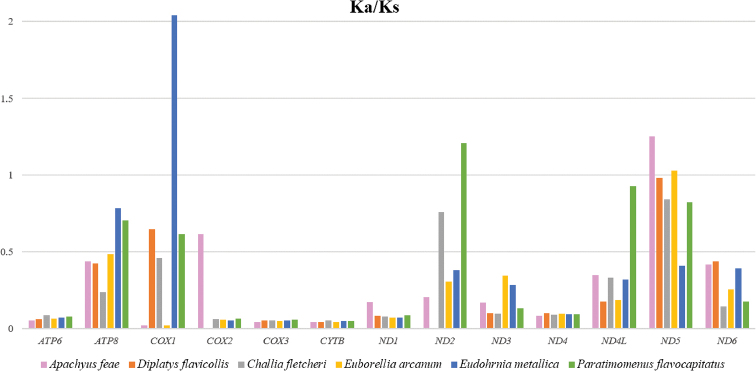
Evolutionary rates of PCGs in six species of earwigs. The bar indicates each gene’s Ka/Ks value.

The ratio of Ka/Ks was calculated for each PCG of the six earwig mitogenomes to evaluate the evolutionary rates of the PCGs (Fig. 4). The results showed that *COX1* of *E.metallica* has the highest evolutionary rate, followed by *ND5* of *A.feae* and *ND2* of *P.flavocapitatus*, whereas *COX1* of *A.feae* and *E.arcanum* appear to be the lowest. The genes with ratios of Ka/Ks above 1 are evolving under positive selection. Other genes with ratios of Ka/Ks below 1 are expected to evolve under purifying selection.

### ﻿Transfer RNA (tRNA) genes

**Figure 5.**
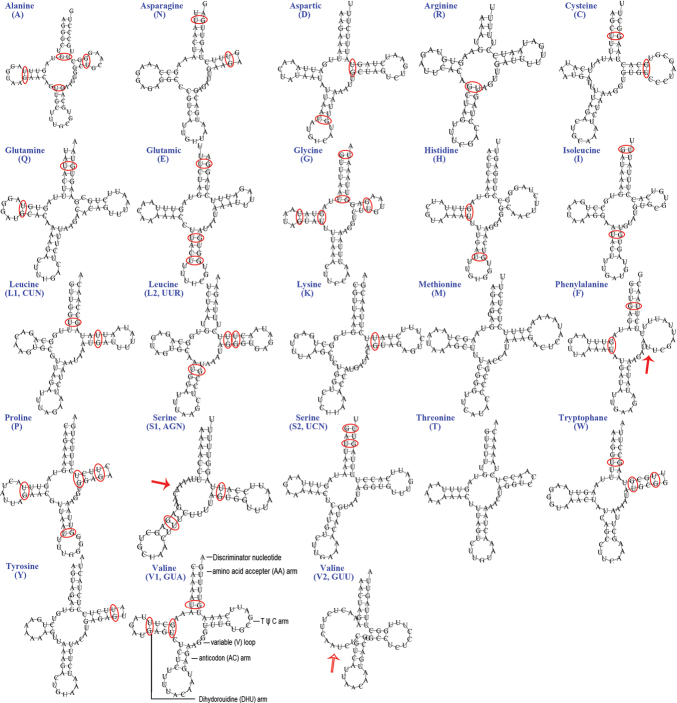
Secondary structures of tRNA genes in the mitogenome of *Apachyusfeae.* Mismatched base pairs are indicated by red circles; reduced arms are indicated by red arrowheads.

**Figure 6.**
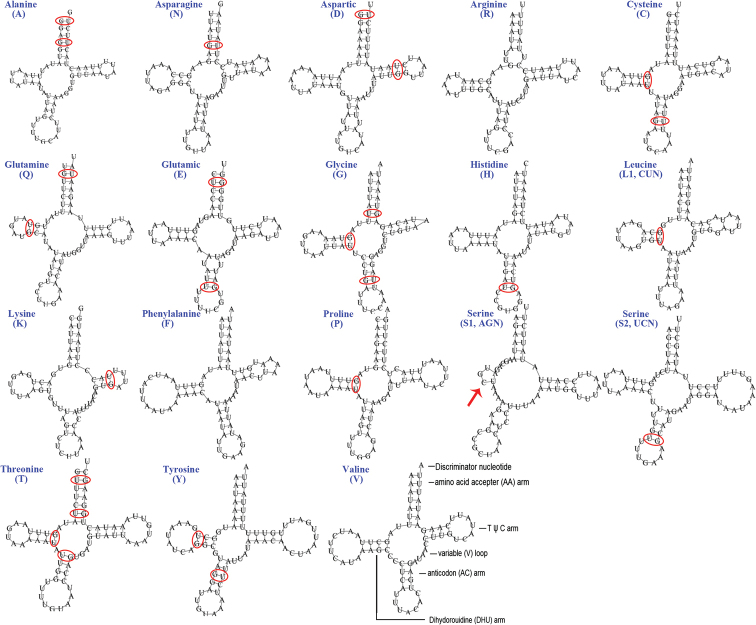
Secondary structures of tRNA genes in the mitogenome of *Diplatysflavicollis.* Mismatched base pairs are indicated by red circles; reduced arms are indicated by red arrowheads.

The typical set of 22 tRNA genes and an extra *trnV* gene are detected in the mitogenome of *A.feae* (Fig. 5). In *D.flavicollis*, 18 tRNA genes are recognized and the four tRNA genes *trnI*, *trnM*, *trnW*, *trnL* are absent due to the incomplete sequencing of 5´ end (Fig. 6). In other sequenced earwigs, *C.fletcheri* has all 22 tRNA genes (Wan et al. 2012), *E.arcanum* lacks *trnY*, and *E.metallica* and *P.flavocapitatus* lack *trnR* and *trnS1*. Individual tRNA gene of the two newly sequenced mitogenomes range in size from 60 to 75 bp; the longest tRNA gene is *trnY* in *D.flavicollis*, and the shortest tRNA gene is the extra *trnV* in *A.feae*. In the mitogenomes of *A.feae* and *D.flavicollis*, most of the tRNA genes exhibit cloverleaf secondary structures, but the dihydrouridine (DHU) arm is lost for the extra *trnV* of *A.feae* and is reduced for *trnS1* of both species. The anticodons of the tRNA genes were identical among the earwigs. In the tRNA genes of *A.feae* and *D.flavicollis*, a total of 48 and 25 mismatched base pairs are respectively recognized and all of them are G-U pairs.

### ﻿Ribosomal RNA (rRNA) genes

Two rRNA genes are consistently found in all sequenced mitogenomes. Locations of the two rRNA genes are conserved among earwig species and similar to *D.yakuba*, but the lengths are variable. In *A.feae*, the large ribosomal RNA (*rrnL*) gene is 1280 bp in length with an A+T content of 67.9%; the small ribosomal RNA (*rrnS*) gene is 739 bp with an A+T content of 66.0%. In *D.flavicollis*, the *rrnL* gene is 1364 bp with an A+T content of 76.1%; the *rrnS* gene is 960 bp with an A+T content of 76.9%.

### ﻿Control region

**Figure 7.**
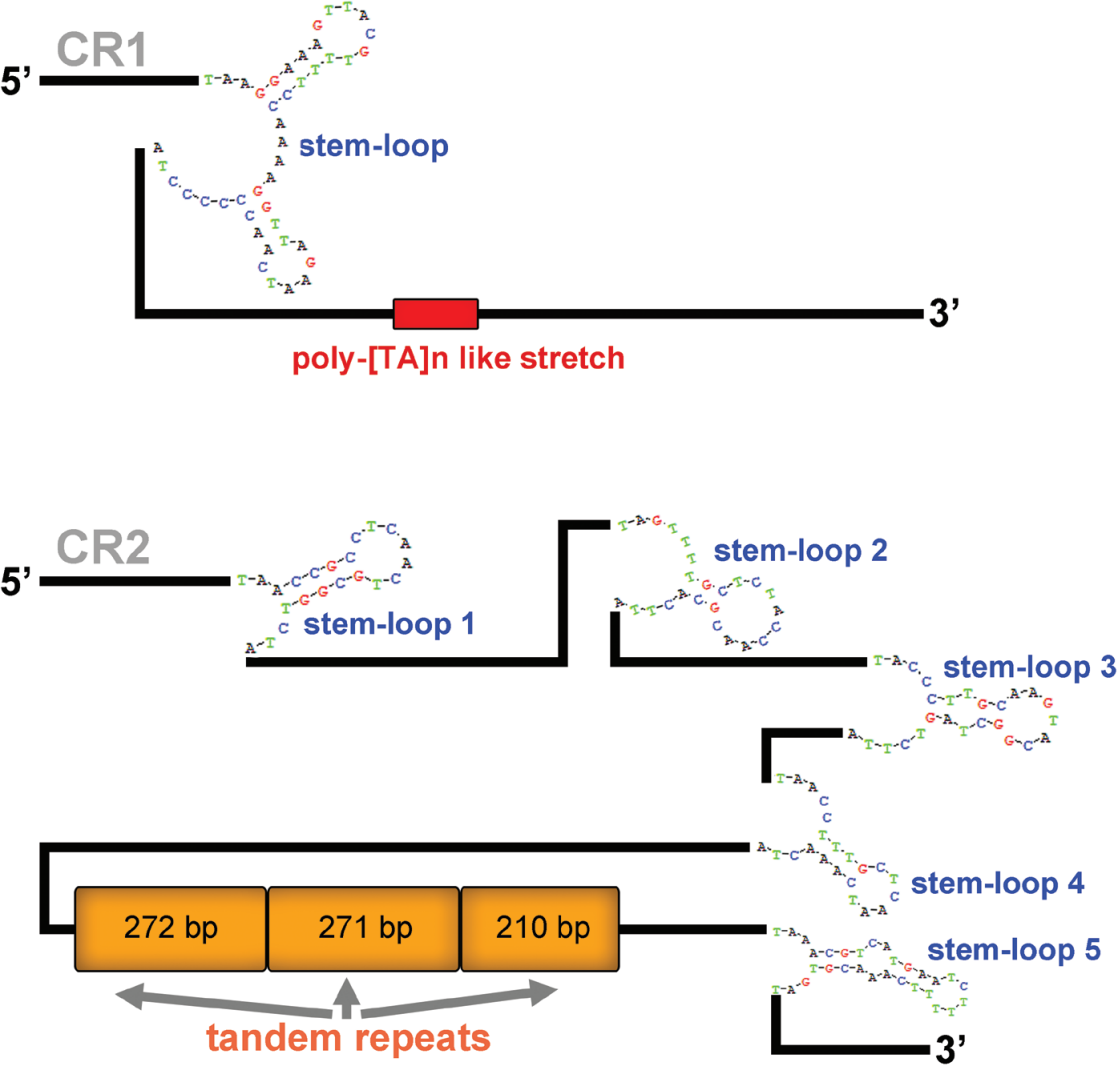
Predicted structural elements in the control regions of *Apachyusfeae*.

**Figure 8.**
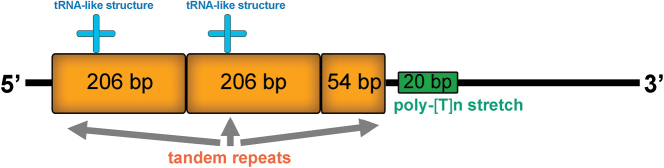
Predicted structural elements in the control region of *Diplatysflavicollis*.

**Figure 9.**
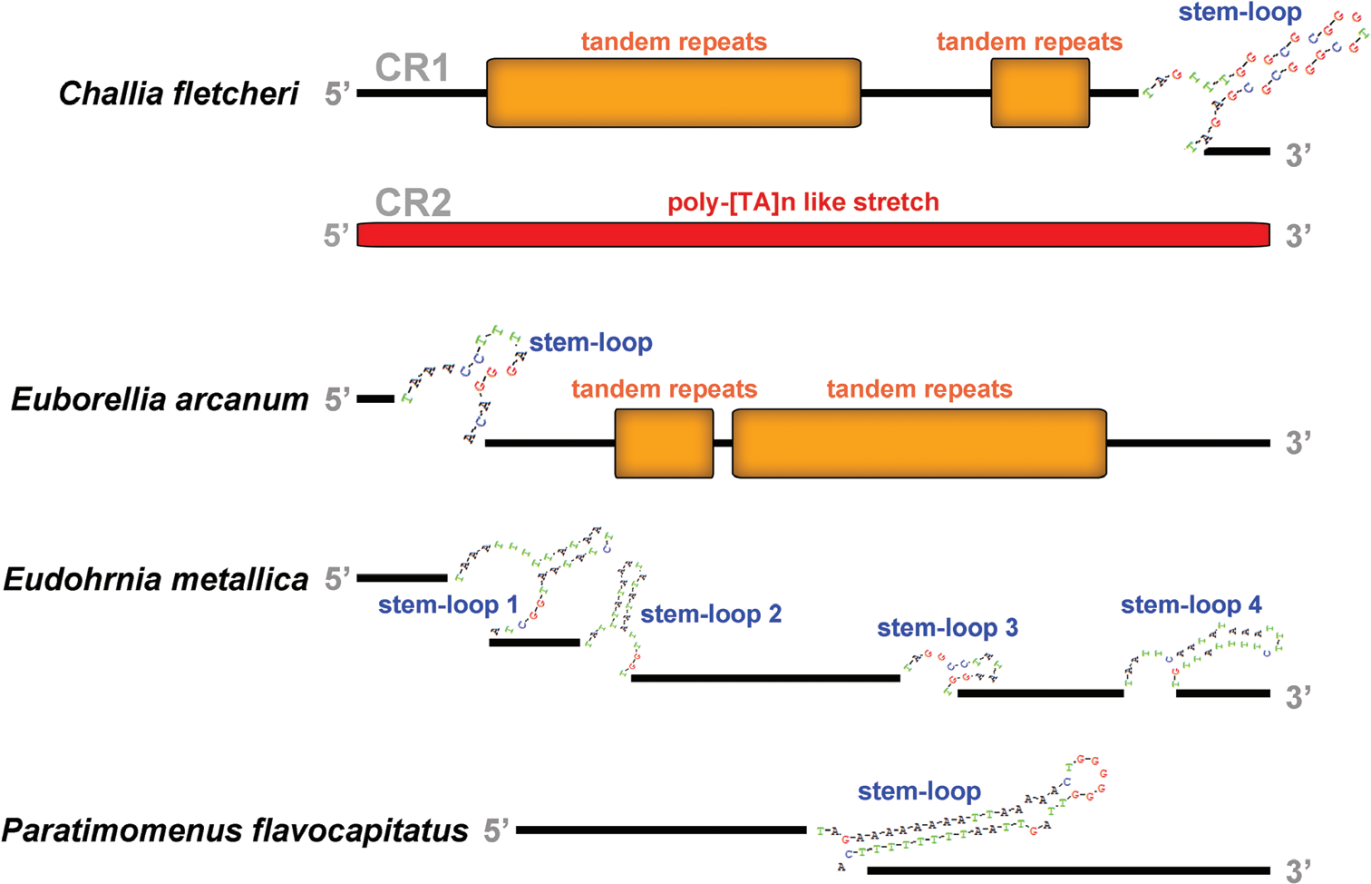
Predicted structural elements in the control regions of *Challiafletcheri*, *Euborelliaarcanum*, *Eudohrniametallica*, and *Paratimomenusflavocapitatus*.

Two putative control regions (CRs) are found in the mitogenomes of *A.feae*, *E.metallica* and *P.flavocapitatus*. The CR1 of *A.feae* is 756 bp and located after *rrnS*, containing a stem-loop (SL) structure and a poly-[TA]n like stretch (Fig. 7). The CR2 of *A.feae* is 3285-bp long and located between *trnS2* (*UCN*) and *ND1*, being composed of five SL structures and three copies of tandem repeats. The CR of *D.flavicollis* is 592 bp and located between *trnQ* and *trnS1*, comprising two and partial copies of tandem repeats, two tRNA-like structures, and a poly-[T]n stretch (Fig. 8). In *C.fletcheri*, the 1816-bp long CR1 contains a SL structure and two regions of tandem repeats; the entire 2856-bp long CR2 comprises 21.1 copies of tandem repeats (Fig. 9). The CR of *E.arcanum* is 686 bp in size, containing a SL structure, a poly-[TA]n stretch and a tandem repeats region (Fig. 9). The 891-bp long CR of *E.metallica* comprises four SL structures (Fig. 9). The CR of *P.flavocapitatus* is short, 227-bp in size, and contains one SL structure (Fig. 9).

### ﻿Phylogenetic analyses

**Figure 10.**
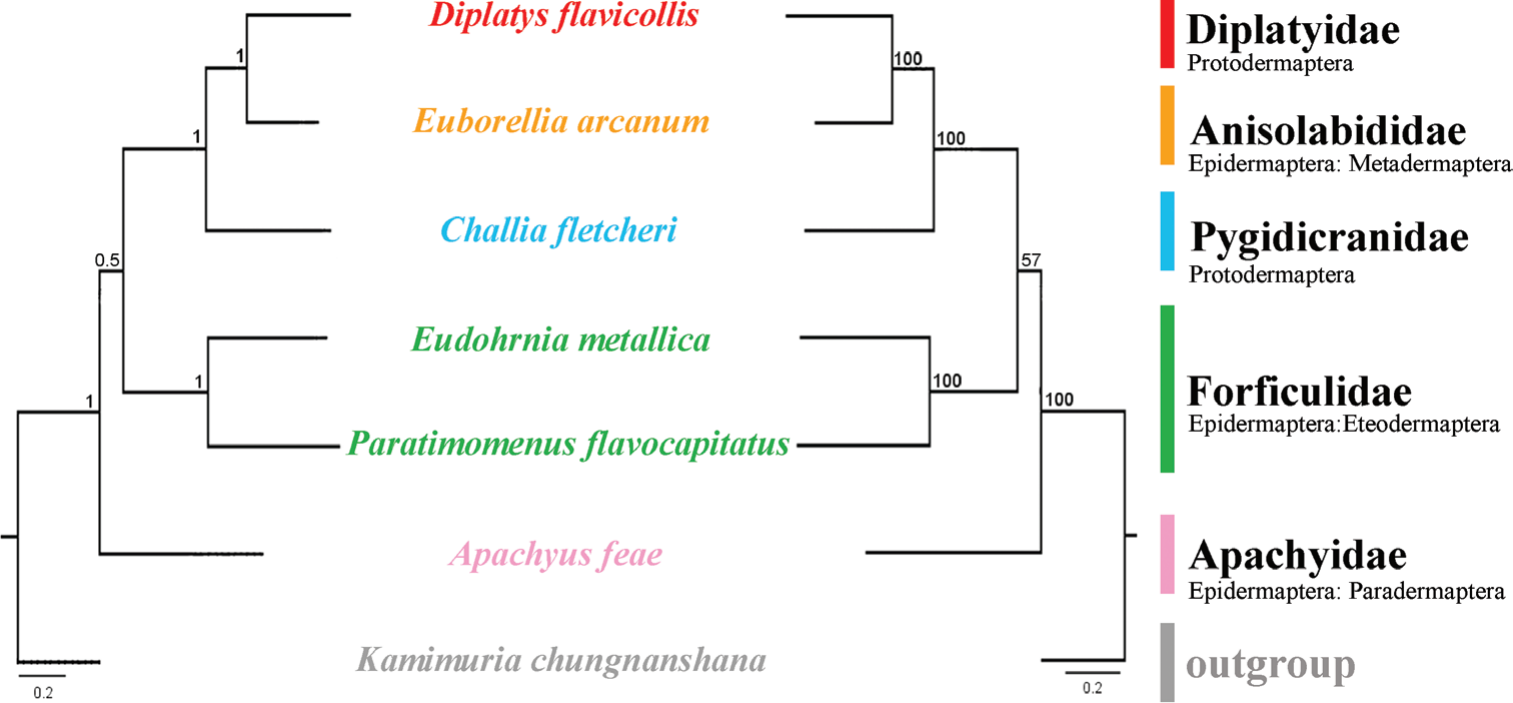
Phylogenetic relationships within Dermaptera inferred by Bayesian inference and maximum likelihood analysis. Numbers at the nodes are posterior probabilities (left) and bootstrap values (right). The family names are listed after the species. Infraorders and parvorders are indicated below each family name.

The phylogenetic analyses use the nucleotide sequences of six available earwig mitogenomes to investigate the mitochondrial phylogenetic relationships within Dermaptera. The two phylogenetic trees using BI and ML analyses generated identical topological structures for Dermaptera (Fig. 10). The monophyly of Forficulidae is supported with high values. Diplatyidae is recovered as the sister group of Anisolabididae and their combined clade is grouped with Pygidicranidae. Apachyidae is supported as the sister group to other sequenced families. Monophyly of the two infraorders Protodermaptera and Epidermaptera cannot be supported by either analysis. The three parvorders Paradermaptera, Metadermaptera, and Eteodermaptera are each represented by single family and their relationship was recovered as Paradermaptera + (Eteodermaptera + Metadermaptera).

## ﻿Discussion

This study sequenced and comparatively analyzed two earwig mitogenomes with other available public data. The mitogenomes of *A.feae* and *D.flavicollis* were slightly smaller in size than that of *C.fletcheri* (20,456 bp) (Wan et al. 2012). Unlike most other insects (Wei et al. 2010), the *A.feae* mitogenome has both negative AT-skew and GC-skew values as in *E.metallica* and *P.flavocapitatus*, whereas *D.flavicollis* exhibits negative AT-skew and positive GC-skew values as in *C.fletcheri* (Wan et al. 2012) and *E.arcanum*. The number of mitochondrial genes and control regions were variable in Dermaptera, either with the addition or loss of several tRNA genes. In other four completely or partially sequenced mitogenomes of Dermaptera, the presence of typical 37 genes and two elongated control regions is found in *C.fletcheri* (Wan et al. 2012), the lack of *trnY* is found in *E.arcanum*, and the absence of *trnR* and *trnS1* (*AGN*) occurs in both *E.metallica* and *P.flavocapitatus*. The presence of an elongated control region or an extra control region is temporarily considered a common phenomenon in earwig mitogenomes. The elongated non-coding regions in Dermaptera (as found in *A.feae* and *C.fletcheri*) could contribute to the frequently large mitogenomic size (Wan et al. 2012), which is also common in other insect orders, such as in Plecoptera (Chen and Du 2017). Multiple IGNs were present in all available mitogenomes of Dermaptera, indicating a loose mitogenomic structure for the earwigs. No PCG rearrangements were found in all sequenced earwigs (Fig. 2). The PCGs and rRNA genes of Dermaptera seemed conserved in arrangements, but this should be confirmed by more mitogenomic data. Rearrangement of tRNA genes were detected in all sequenced earwig species (Fig. 2). The rearrangements concerning tRNA genes occur very frequently in the sequenced earwigs and mainly focus on the *trnA*-*R*-*N*-*S1*-*E*-*F* gene cluster, which is similar to the arrangement in Lepidoptera (Cao et al. 2012; Gong et al. 2012; Wang et al. 2014; Park et al. 2016). Extensive mitochondrial rearrangement events are expected to occur in other unsequenced earwigs.

The Ka/Ks calculation revealed the fast-evolving *COX1* and slow-evolving *CYTB* in earwigs. The fast-evolving genes are potential candidates as molecular markers for future genetic studies of Dermaptera. Among the very few molecular studies of Dermaptera, Naegle et al. (2016), Stuart et al. (2019), and Kirstová et al. (2020) supported the efficiency of *COX1* gene in species delimitation and phylogenetic reconstruction. In tRNA genes, reductions of *trnS1* DHU arms was very common in other metazoans (Garey and Wolstenholme 1989). The shortened DHU arm of *trnS1* found in *A.feae* and *D.flavicollis* was also found in *C.fletcheri* but absent in other earwigs (Wan et al. 2012).

The control regions of Dermaptera were highly variable in size, location, and secondary structures. The putative structural elements in the CRs included SL structure, poly-[TA]n like stretch, tandem repeats, tRNA-like structure and poly-[T]n stretch, and they were highly variable in both size and numbers, which implied that the earwig mitogenomes are likely to be regulated in apparent different ways during the mitogenomic replication and transcription processes.

In the phylogenetic analyses, the monophyly of Forficulidae was supported with high values The basal phylogenetic position of Apachyidae was also recovered based on nuclear single-copy genes (Wipfler et al. 2020). However, the current relationship between the five earwig families is entirely incongruent with all previous phylogenetic studies using either morphological data, other types of molecular markers, or combined data (Haas 1995; Guillet and Vancassel 2001; Haas and Kukalova-Peck 2001; Colgan et al. 2003; Jarvis et al. 2005; Kocarek et al. 2013; Naegle et al. 2016; Wipfler et al. 2020). The preliminary phylogenetic analyses in current study included very few representatives from only five earwig families and thus insufficient for comparison with previous studies. The currently available mitogenomic data could not resolve the relationship within Dermaptera. More comprehensive sampling and sequencing work are necessary to clarify the mitogenomic features and mitogenomic phylogeny of Dermaptera.

## ﻿Conclusions

The mitochondrial genomes of *A.feae* and *D.flavicollis* were sequenced, analyzed, and compared with other sequenced earwigs. The phylogenetic reconstructions with BI and ML methods generated identical topology but differed from previous phylogenetic studies using morphological data or other molecular markers. Due to the limited sample size, the relationships found here must be treated with caution. More mitogenomes should be obtained in future works to resolve the phylogeny of earwigs.
